# Molecular mechanisms by which HERV-K Gag interferes with HIV-1 Gag assembly and particle infectivity

**DOI:** 10.1186/s12977-017-0351-8

**Published:** 2017-04-26

**Authors:** Kazuaki Monde, Hiromi Terasawa, Yusuke Nakano, Ferri Soheilian, Kunio Nagashima, Yosuke Maeda, Akira Ono

**Affiliations:** 1 Department of Medical Virology, Graduate School of Medical Sciences, Faculty of Life Sciences, Kumamoto University, Kumamoto, Japan; 2 Department of Microbiology, Faculty of Life Sciences, Kumamoto University, Kumamoto, Japan; 3 Department of Microbiology and Immunology, University of Michigan Medical School, Ann Arbor, MI USA; 4 Electron Microscopy Laboratory, Leidos Biomedical Research, Inc, Frederick National Laboratory for Cancer Research, Frederick, MD USA; 5 Laboratory of Viral Pathogenesis, Institute for Virus Research, Kyoto University, Kyoto, Japan

**Keywords:** HIV-1, HERV-K, Gag coassembly

## Abstract

**Background:**

Human endogenous retroviruses (HERVs), the remnants of ancient retroviral infections, constitute approximately 8% of human genomic DNA. Since HERV-K Gag expression is induced by HIV-1 Tat in T cells, induced HERV-K proteins could affect HIV-1 replication. Indeed, previously we showed that HERV-K Gag and HIV-1 Gag coassemble and that this appears to correlate with the effect of HERV-K Gag expression on HIV-1 particle release and its infectivity. We further showed that coassembly requires both MA and NC domains, which presumably serve as scaffolding for Gag via their abilities to bind membrane and RNA, respectively. Notably, however, despite possessing these abilities, MLV Gag failed to coassemble with HIV-1 Gag and did not affect assembly and infectivity of HIV-1 particles. It is unclear how the specificity of coassembly is determined.

**Results:**

Here, we showed that coexpression of HERV-K Gag with HIV-1 Gag changed size and morphology of progeny HIV-1 particles and severely diminished infectivity of such progeny viruses. We further compared HERV-K-MLV chimeric constructs to identify molecular determinants for coassembly specificity and for inhibition of HIV-1 release efficiency and infectivity. We found that the CA N-terminal domain (NTD) of HERV-K Gag is important for the reduction of the HIV-1 release efficiency, whereas both CA-NTD and major homology region of HERV-K Gag contribute to colocalization with HIV-1 Gag. Interestingly, these regions of HERV-K Gag were not required for reduction of progeny HIV-1 infectivity.

**Conclusions:**

Our results showed that HERV-K Gag CA is important for reduction of HIV-1 release and infectivity but the different regions within CA are involved in the effects on the HIV-1 release and infectivity. Altogether, these findings revealed that HERV-K Gag interferes the HIV-1 replication by two distinct molecular mechanisms.

**Electronic supplementary material:**

The online version of this article (doi:10.1186/s12977-017-0351-8) contains supplementary material, which is available to authorized users.

## Background

Long terminal repeat (LTR)-bounded elements, which are called human endogenous retroviruses (HERVs), comprise about 8% of the human genome [1–3]. HERVs have infected germ lineage cells, and therefore their proviruses are transmitted vertically from ancestors to progeny in human genomic DNA [4]. During a period exceeding a million years, they have acquired numerous mutations or deletions and therefore no longer encode infectious retrovirus [5]. HERV-K, which is relatively new endogenous retrovirus among HERV families, apparently contains a set of intact open reading frames [6]. However, all known HERV-K proviruses are replication incompetent [7–9]. Two groups reconstructed infectious HERV-K sequences by aligning full-length HERV-K proviruses [9, 10]. The infectious HERV-K clones have become a widely used tool for biological research of HERV-K.

Virion assembly of HIV-1 as well as HERV-K occurs at the plasma membrane (PM) [9, 11]. HIV-1 Gag consists of four major domains, matrix (MA), capsid (CA), nucleocapsid (NC) and p6 [12]. These domains mediate each step of the virion assembly events. The MA domain promotes Gag targeting and binding to the PM. The CA domain mediates the Gag–Gag interactions for assembly of the immature virion and formation of conical shell of the mature viral core. The NC domain binds the viral genome through the two zinc finger motifs and facilitates Gag multimerization during viral assembly. p6, which contains late domain motifs, binds TSG101 and ALIX, recruits the ESCRT machinery and facilitates release of nascent virus particles from the PM [13–15]. Similar to HIV-1 Gag, HERV-K Gag consists of 4 major domains, MA, CA, NC and p15 [16, 17]. The N-terminus of the MA domain is likely myristoylated and essential for Gag binding to the PM [18]. The CA domain contains the major homology region (MHR) that is conserved among retroviruses [16]. The NC domain encodes two zinc finger motifs for RNA binding [16, 17]. The late domain in p15 is essential for the pinch-off of virus from the PM [19]. Unlike the p6 domain of HIV-1 Gag, p15 is located between MA and CA in HERV-K Gag. The functions of each domain of HERV-K Gag are likely to be similar to those of HIV-1 Gag [16–20].

All human cells harbor HERV-K genomes. HERV-K is expressed in germ cells and under some pathological conditions [21–25]. In HIV-1-infected patients, antibodies and T cell responses against HERV-K are detected [26–30]. Furthermore, HERV-K RNA and Gag protein are upregulated in plasma samples of HIV-1-infected patients [28, 31–36]. It has been also shown that HIV-1 Tat changes the state of heterochromatin and induces the HERV-K expression in somatic cells [37–39]. Thus, it is possible that HERV-K Gag exists simultaneously with HIV-1 Gag in same host cells. In our previous study, we observed that HERV-K Gag of the reconstructed clone coassembles with HIV-1 Gag at the PM when overexpressed [18]. For the coassembly of HERV-K Gag with HIV-1 Gag, membrane binding via MA domains and RNA binding via NC domains are essential. Importantly, the virus release efficiency and infectivity of HIV-1 were substantially reduced when coassembly of HIV-1 Gag with HERV-K Gag was observed.

Previously, we showed that HIV-1 Gag coassembles with HERV-K Gag but not MLV Gag [18]. However, MLV Gag binds the PM and RNA like HERV-K Gag. Therefore, it is likely that there is an unknown mechanism that determines the specificity of interaction between HERV-K Gag and HIV-1 Gag. In this study, we determined the domains responsible for the specific interaction between HERV-K Gag and HIV-1 Gag. We found that HERV-K Gag CA MHR promotes specific colocalization with HIV-1 Gag most efficiently, whereas HERV-K Gag CA N-terminal domain (NTD) is needed for HERV-K-Gag-mediated interference of HIV-1 release. However, both HERV-K Gag CA-MHR and CA-NTD were not required for reduction of HIV-1 infectivity, which coincided with a change in the size of HIV-1 particles. Together, these data provide the two distinct molecular mechanisms for the HERV-K Gag interference against HIV-1 replication.

## Methods

### Plasmids

For expression of HERV-K Gag, pCRVI/HERV-K/Gag, a kind gift from P. Bieniasz, was used in this study [9]. This plasmid encodes the HERV-K_CON_ Gag sequence following a CMV promoter and a sequence corresponding to the HIV-1 5′ untranslated region (nt 428–785 in pNL4-3), along with ORFs encoding HIV-1 Rev, Tat, and Vpu. pCRVI/HERV-K/Gag-Flag, pCRVI/HERV-K/Gag-Venus, pCRVI/HIV-1/Gag-Flag, pCRVI/HIV-1/Gag-Venus, pCRVI/HIV-1/Gag-mRFP, pCRVI/MLV/Gag-Flag, and pCRVI/MLV/Gag-Venus were described previously [18]. Chimeric HERV-K Gag constructs were derived from the pCRVI/HERV-K Gag-Flag and pCRVI/MLV Gag-Flag. TSG101-DN was constructed in the same design as Tsg-5’ described previously [40]. HIV-1/YP(−) was created by PCR mutagenesis and contains amino acid substitutions in the ALIX binding motif (YP).

### Cells

HeLa cells and TZM-bl cells were cultured in Dulbecco’s modified Eagle’s medium (DMEM) (Sigma) supplemented with 5% FBS (DMEM-10). TZM-bl (also called JC.53.bl-13) is a HeLa cell derivative that stably expresses large amounts of CD4 and CCR5 [41]. TZM-bl cells that harbor Tat-responsive reporter genes for firefly luciferase (Luc) and *Escherichia coli* B-galactosidase were obtained through the AIDS Research and Reference Reagent Program, Division of AIDS, NIAID, NIH from Dr. John C. Kappes, Dr. Xiaoyun Wu and Tranzyme Inc [42].

### p24 ELISA

HeLa cells were cotransfected with pNL4-3 and indicated pCRVI plasmids using lipofectamine 2000 (Invitrogen) according to the manufacturer’s instructions. At 16 h post-transfection, the supernatants were filtered through 0.45-μm filters, and virions in the supernatants were pelleted down by ultracentrifugation (83,500×*g*, 4 °C, 45 min). Gag proteins in the virion lysates were quantified by p24 ELISA according to the manufacturer’s instruction (MBL).

### Virus release assay

HeLa cells were cotransfected with pNL4-3 and indicated pCRVI plasmids. At 16 h post-transfection, virions in the supernatants were collected and pelleted down by ultracentrifugation (83,500×*g*, 4 °C, 45 min). Cells and virions were lysed with 0.5% TritonX lysis buffer [50 mM Tris–HCl pH7.5 containing 0.5% TritonX-100, 300 mM NaCl, 10 mM Iodoacetamide with protease inhibitor cocktail (Roche)]. Gag proteins in the cell and virion lysates were detected by immunoblotting using HIV-Ig (NIH AIDS Research and Preference Reagent Program), mouse monoclonal anti-Flag antibody (Wako), mouse monoclonal anti-HERV-K Gag antibody (HERM-1831-5) (Austral Biologicals) as primary antibodies. HRP-conjugated anti-human Ig antibodies (Jackson ImmunoResearch) and anti-mouse Ig (Amersham) were used as a secondary antibody. Detection using a HRP-conjugated secondary antibody was performed using the Chemi-Lumi One L (Nacalai tesque).

### Rate-zonal gradient analysis

Rate-zonal gradient analysis was performed as previously described [43]. Virions in cell-free supernatants were collected and centrifuged at a low speed (8000×*g*, 5 min) to remove cellular debris. Virions were pelleted by ultracentrifugation and re-suspended in 1 ml of RPMI-10. Each concentrated sample was layered onto 10–30% sucrose and ultracentrifuged (83,500×*g*, 4 °C, 45 min) in a Beckman SW41Ti. Then 1 ml fractions were collected from each gradient. Amounts of HIV-1 Gag proteins in each fraction were measured by p24 ELISA.

### Infectivity analysis of virions in each sucrose fraction

Each sucrose fraction was two-fold diluted with RPMI-10. For removing sucrose from each fraction, virions in each fraction were pelleted down by ultracentrifugation and resuspended with RPMI-10. Amounts of virions were determined by p24 ELISA.

For analysis of virus infectivity using TZM-bl cells, 3 × 10^4^ cells were inoculated with virus stocks normalized by the amount of p24 Gag (2 ng of p24 Gag) for 2 h. Two days post-infection, Luc activities in TZM-bl cells were measured according to the manufacturer’s instruction (Promega).

### Fluorescence microscopy

HeLa cells were plated in 8-well chamber slides (Nunc) 1 day before transfection at 3.0 × 10^4^ cells/well. At 16 h post-transfection, HeLa cells cotransfected with plasmids encoding YFP- and mRFP-tagged Gag proteins were fixed with 4% paraformaldehyde (Wako) in PBS for 30 min at room temperature, washed once with PBS, and mounted in Fluoromount-G (Dako). The images of 20–50 fields were recorded using a Zeiss LSM 700 laser-scanning confocal microscopy. Colocalization between YFP- and mRFP-tagged Gag was quantified using the ZEN software (Zeiss) with which we calculate the Pearson correlation coefficient (R-value). We set the entire cell body of each YFP- and mRFP-coexpressing cell as the region of interest for this analysis. R = 1 represents perfect co-localization, and R = 0 represents random distributions of fluorescence intensities.

### Transmission electron microscopy analysis

HeLa cells were transfected with indicated plasmids. Cells were fixed 16 h post-transfection with 2% glutaraldehyde in PBS. Cells were analyzed on a Hitachi H7600 transmission electron microscope as previously described [44].

## Results

### Coassembly of HERV-K Gag alters HIV-1 particle properties

**Fig. 1:**
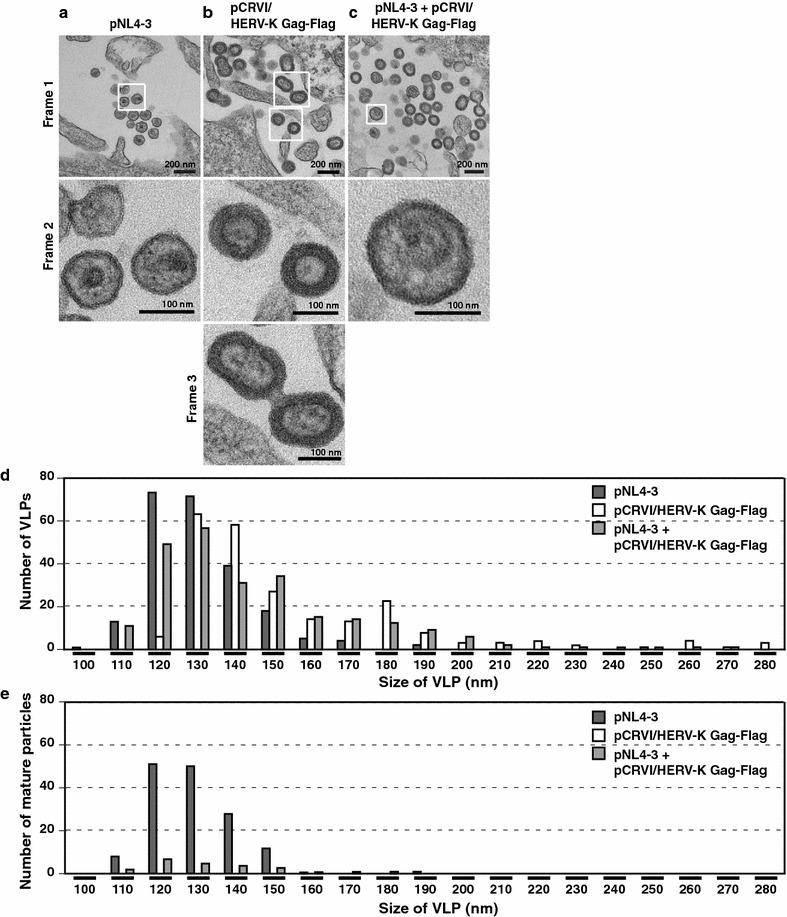
Coassembly of HERV-K Gag changes properties of HIV-1 particles. HeLa cells expressing HIV-1 molecular clone (**a**), HERV-K Gag-FLAG (**b**), or HIV-1 molecular clone and HERV-K Gag-FLAG (**c**) were analyzed by transmission electron microscopy at 16 h post-transfection. *Bars* in frame 1, 200 nm; *bars* in frame 2 and 3, 100 nm. **d**, **e** The sizes of VLPs or mature particles, which are released from transfected cells, were measured using images of transmission electron microscopy. Total numbers of VLPs examined are 232, 239, and 256 for cultures expressing HIV-1 alone, HERV-K Gag-FLAG alone, and HIV-1 and HERV-K Gag-FLAG, respectively

We previously reported that ectopic expression of HERV-K Gag causes coassembly of HIV-1 Gag and HERV-K Gag and reduces relative infectivity of the progeny HIV-1 virions [18]. It appears likely that coassembly of HERV-K Gag with HIV-1 Gag would disturb the organization of Gag lattice that eventually forms HIV-1 particles. To determine whether HERV-K Gag changes the morphology of HIV-1 particles, we performed transmission electron microscopy analyses using HeLa cells transfected with plasmids expressing HIV-1 and HERV-K Gag. We found that particle sizes were different between HIV-1 and HERV-K (Fig. 1a, b). HIV-1 particles were slightly smaller than HERV-K particles (Fig. 1d). Average sizes of HIV-1 and HERV-K particles were 131.6 and 156.1 nm, respectively. The production of mostly immature HERV-K particles as shown in Fig. 1b (particles with no obvious electron-dense cores) is likely due to the lack of HERV-K protease. Unlike HIV-1 particles, HERV-K particles appeared to form both single and connected particles of doublet or more (Fig. 1b), both of which have been also shown in reports published by other groups [9, 21]. In the presence of both HIV-1 Gag and HERV-K Gag, particles appeared larger than HIV-1 particles produced in the absence of HERV-K Gag (Fig. 1a, c). Indeed, the average size of particles produced by the cotransfected cells was 146.1 nm (Fig. 1d). Moreover, mature HIV-1 particles are rare in the presence of HERV-K Gag (Fig. 1c). The number of mature particles was 151 out of 232 in the absence of HERV-K Gag, whereas the mature particle number was only 24 out of 256 in the presence of HERV-K Gag (Fig. 1e). The extent of reduction in mature particle numbers in cotransfected cultures relative to the cultures transfected with HIV-1 alone was constant in both 110–120 and 130–140 nm ranges of particle size. Since HERV-K Gag produces VLPs with the size typically in the latter but not the former range (Fig. 1d), it is unlikely that mature HIV-1 particles appeared rare just because HIV-1 particles were a minor population compared to HERV-K particles in the cotransfected cultures (Fig. 1e). These results suggest that coassembly of HERV-K Gag increases the number of HIV-1 particles with aberrant sizes and/or a maturation defects, which correlates with the previously observed reduction in the HIV-1 infectivity.

**Fig. 2:**
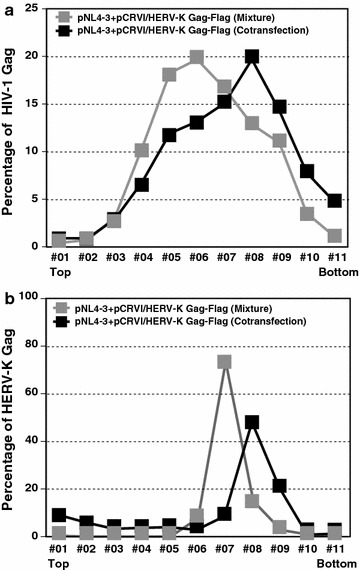
Coassembly with HERV-K Gag changes the size of particles containing HIV-1 p24 Gag. HeLa cells were separately transfected or cotransfected with the HIV-1 molecular clone pNL4-3 and indicated plasmids. For analysis of particles containing single Gag (*gray square*), the supernatants from separately transfected HeLa cells were collected and pooled. For coassembled particles (*black square*), the supernatant of cotransfected HeLa cells was used. These viruses were fractionated in rate-zonal gradient analysis. The amounts of HIV-1 Gag were measured by ELISA (**a**), and the amounts of HERV-K Gag were measured by immunoblotting using anti-Flag antibody (**b**). Representative data from five independent experiments are shown

To validate the observed size difference using a different method, we fractionated the different size of particles in 10–30% rate-zonal sucrose gradients. In stokes’ law, larger particles would precipitate faster than smaller particles. In these experiments, we observed that the peak of HIV-1 Gag p24 appeared in a middle fraction (Fraction #06) (Fig. 2a) that is slightly higher than the peak of HERV-K Gag (Fraction #07) (Fig. 2b). These results indicate that HERV-K particles are indeed slightly larger than HIV-1 particles. When HIV-1 Gag and HERV-K Gag were coexpressed, we observed that the peaks of both HIV-1 Gag p24 and HERV-K Gag shifted to the lower fraction (Fraction #08) than when singly expressed (Fig. 2a, b). These results indicate that coassembly of HERV-K Gag changes the size or density of HIV-1 virions. Altogether, the electron microscopy and gradient results suggest that HERV-K Gag changes the properties of HIV-1 virions via coassembly, which may explain the observed reduction in infectivity.

### HERV-K Gag CA-NTD is required for reduction of HIV-1 release

**Fig. 3:**
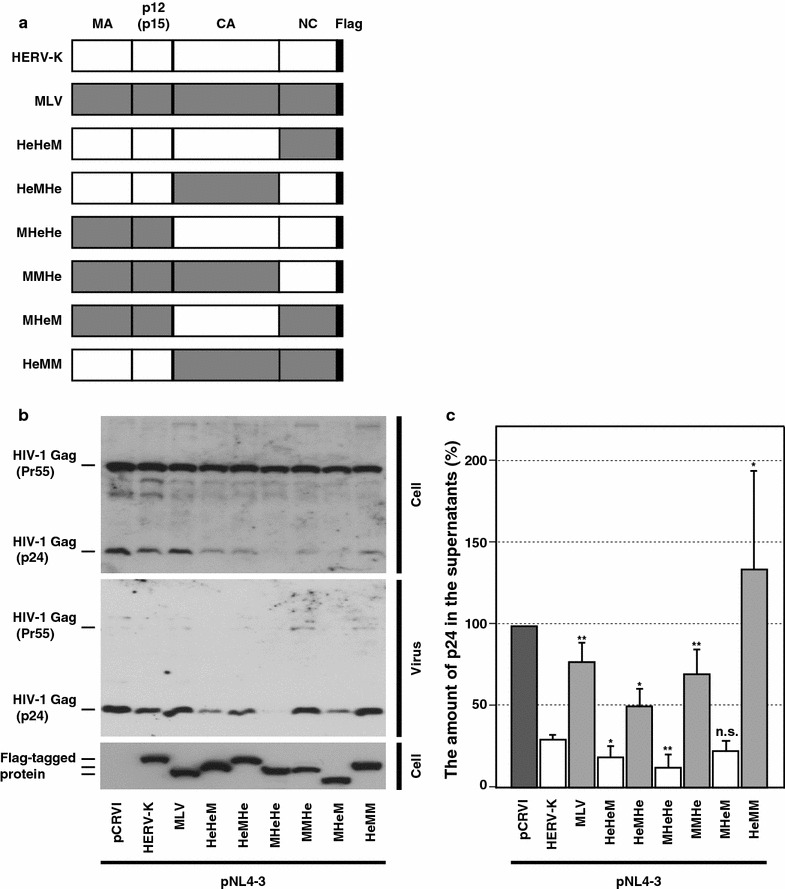
Coexpression of Gag chimeras containing HERV-K CA reduces HIV-1 release efficiency. **a** pCRVI/HeHeM, HeMHe, MHeHe, MMHe, MHeM and HeMM encode chimeras between HERV-K Gag and MLV Gag. All chimeric Gag constructs are tagged with the Flag epitope. **b** Cell and viral lysates from cotransfected cells were subjected to SDS-PAGE and analyzed by immunoblotting with HIV-Ig or anti-Flag antibody. **c** HeLa cells were cotransfected with the HIV-1 molecular clone pNL4-3 and indicated plasmids. The amount of p24 in the virus pellet was measured by ELISA. An empty vector pCRVI was used as a control. pCRVI/HERV-K Gag-Flag and pCRVI/MLV Gag-Flag express Flag-tagged HERV-K Gag and MLV Gag, respectively. Data from three independent experiments are shown as means ± standard deviations

HERV-K Gag coassembles with HIV-1 Gag and reduces not only HIV-1 infectivity but also HIV-1 release efficiency [18]. We confirmed this effect in a broad range of the ratios for plasmids expressing HERV-K and HIV-1 and additionally observed that the presence of HERV-K pro or pol sequence does not affect the HERV K Gag-mediated reduction of HIV-1 infectivity or HIV-1 release efficiency (Additional file 1: Figure S1A–S1D). The reduction of HIV-1 release requires both HERV-K Gag MA and NC domains [18]. These findings suggested that MA-mediated Gag-membrane binding and NC-mediated Gag-RNA binding are important for interaction between HERV-K Gag and HIV-1 Gag. However, MLV Gag, which also binds to membrane via MA and RNA via NC like HERV-K Gag, did neither interact with HIV-1 Gag nor reduce the HIV-1 release. Therefore, it is likely that there is a mechanism that distinguishes HERV-K and MLV Gag during coassembly with HIV-1 Gag and inhibition of HIV-1 release. To determine regions of HERV-K Gag responsible for the specific coassembly with HIV-1 Gag, we designed chimeric constructs consisting of HERV-K Gag and MLV Gag domains (Fig. 3a). These constructs were cotransfected with an HIV-1 molecular clone into HeLa cells. Amounts of plasmids for transfection were adjusted to render similar expression levels of Gag chimeras in HeLa cells, and under this condition virus release was compared by immunoblotting (Fig. 3b). In another set of experiments, we examined the amounts of p24 released into the supernatants of cells cotransfected in the same way using p24 ELISA (Fig. 3c). These experiments showed that HIV-1 release was reduced by ectopic expression of HERV-K Gag but not by expression of MLV Gag as observed previously (Fig. 3b, c). We found that Gag chimeras containing HERV-K CA reduced HIV-1 release efficiency as WT HERV-K Gag. In contrast, MLV CA-containing Gag chimeras did not reduce HIV-1 release efficiently even when they contain the HERV-K MA and NC domains (Fig. 3b, c). These results indicate that the CA domain of HERV-K Gag is required for reduction of HIV-1 release.

**Fig. 4:**
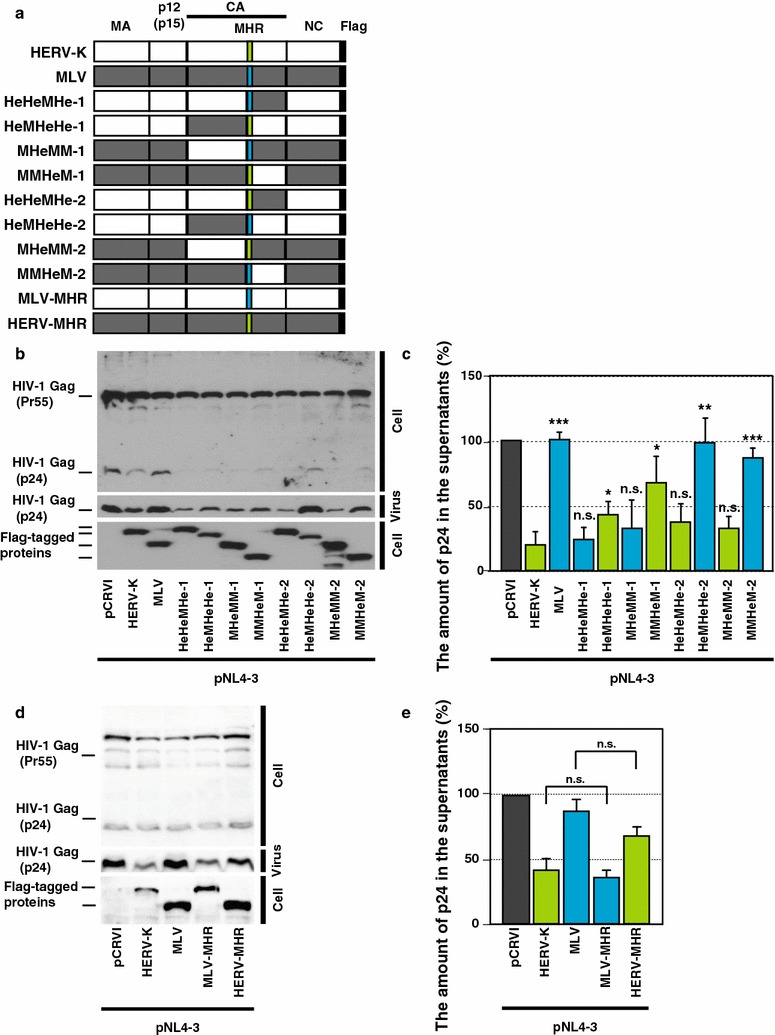
Coexpression of Gag chimeras containing HERV-K CA-NTD reduces HIV-1 release efficiency. **a** pCRVI/HeHeMHe-1, HeMHeHe-1, MHeMM-1, MMHeM-1, HeHeMHe-2, HeMHeHe-2, MHeMM-2 and MMHeM-2 encode CA-domain chimeras between HERV-K Gag and MLV Gag. HERV-K MHR and MLV MHR are shown in *green* and *blue*, respectively. This *same color coding* is used to indicate constructs containing the different MHRs in the subsequent panels in Figs. 4, 8, and 9. **b**, **d** Cell and viral lysates from cotransfected cells were subjected to SDS-PAGE and analyzed by immunoblotting with HIV-Ig or anti-Flag antibody. **c**, **e** HeLa cells were transfected with pNL4-3 and indicated plasmids. The amount of p24 was measured as described in Fig. 3c. *P* values, compared with HERV-K Gag, were determined using a Student’s *t* test. *P < 0.01; **P < 0.001; ***P < 0.0001; *n.s.* not significant

We further analyzed HERV-K CA regions responsible for suppression of HIV-1 release using CA-NTD and -CTD chimeras (Fig. 4a). A MHR which is conserved among retroviruses, is also present at the N-terminal part of the HERV-K Gag CA-CTD [16]. Therefore, we also constructed CA chimeras that contain the NTD and the MHR derived from the same Gag protein (Fig. 4a, denoted by “-2”) and compared their effects on HIV-1 release as done in Fig. 3. HIV-1 release was similarly reduced by coexpression of chimeras HeHeMHe-1, MHeMM-1, HeHeMHe-2 and MHeMM-2 compared to coexpression of WT HERV-K Gag. In contrast, coexpression of HeMHeHe-1, MMHeM-1, HeMHeHe-2 and MMHeM-2 was less efficient in suppressing HIV-1 release compared to that of WT HERV-K Gag (Fig. 4b, c). These results indicate that HERV-K CA-NTD is required for efficient reduction of HIV-1 release. Notably, HIV-1 release was impaired by HeMHeHe-1 but not by HeMHeHe-2 (Fig. 4b, c). However, CA-MHR itself did not affect the HIV-1 release efficiency (Fig. 4d, e). Considering these results and the moderate suppression of HIV-1 release by MMHeM-1, HERV-K CA-MHR in the context of HERV-K CA-CTD seems to cause moderate suppression of HIV-1 release regardless of NTD. Altogether, these results suggest that HERV-K CA-NTD is required for reduction of HIV-1 release and that the combination of HERV-K CA-MHR and -CTD may contribute to, but is not essential for, suppression of HIV release by HERV-K Gag.

### HERV-K Gag reduces the HIV-1 assembly at the early steps

**Fig. 5:**
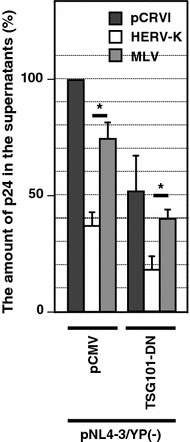
HERV-K Gag reduces release efficiency of HIV-1 at a stage prior to particle pinching-off. HeLa cells were cotransfected with HIV-1/YP(−) and indicated pCMV plasmids and pCRVI plasmids. The amount of p24 in the virus pellet was measured by ELISA

To investigate the mechanism by which HERV-K Gag reduces the HIV-1 release, we used dominant-negative TSG101 (TSG101-DN), which inhibits ESCRT-mediated pinching off of HIV-1 particles from the PM [45]. We used HIV-1/YP(−) to suppress ESCRT recruitment via ALIX so as to specifically examine the TSG101-dependent HIV-1 release. HERV-K Gag further reduced the HIV-1 release about 50% both in the presence and absence of TSG101-DN. This result indicates that HERV-K Gag is likely to inhibit the stage separable from and hence earlier than the pinching off of HIV-1 particles (Fig. 5).

**Fig. 6:**
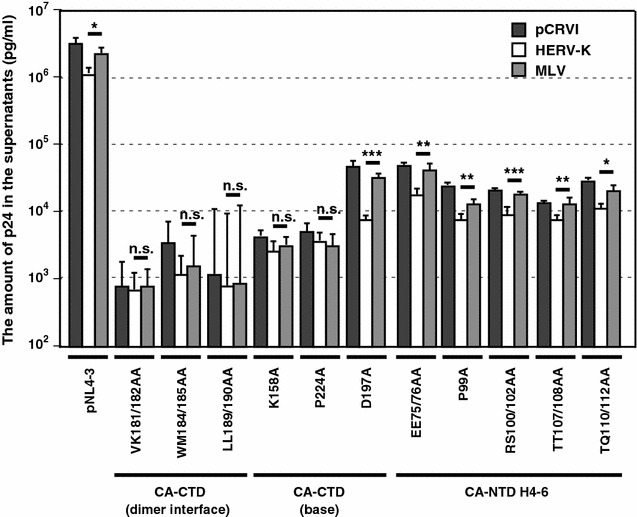
HERV-K Gag reduces release efficiency of HIV-1 through inhibition of an early stage. HIV-1 mutants are defective for the HIV-1 assembly at the early stage. HeLa cells were cotransfected with indicated HIV-1 mutants and indicated pCRVI plasmids at 10:1 ratio. Two days later, the amount of p24 released into the supernatants was measured by ELISA. Data from three independent experiments are shown as means ± standard deviations. *P* values were determined using a Student’s *t* test. *P < 0.01; **P < 0.001; ***P < 0.0001; *n.s.* not significant

To examine which HIV-1 assembly step HERV-K Gag inhibits, we used HIV-1 mutants EE75/76AA, P99A, RS100/102AA, TT107/108AA, TQ110/112AA, VK181/182AA, WM184/185AA, LL189/190AA, K158A, D197A and P224A. HIV-1 Gag containing mutations VK181/182AA, WM184/185AA and LL189/190AA are defective in forming CA-CTD dimer [46–52]. Recent studies [52] suggest that CA-CTD base mutations K158A, D197A and P224A destabilize the first membrane-targeted assembly intermediate, while the CA-CTD dimer interface residues (V181, K182, W184, M185, L189, L190) are required for continued multimerization of Gag at the PM. These and other studies also showed that mutations EE75/76AA, P99A [53, 54], RS100/102AA, TT107/108AA and TQ110/112AA are defective in the final step of HIV-1 particle assembly. We found that the release efficiency of HIV-1 mutants, EE75/76AA, P99A, RS100/102AA, TT107/108AA, TQ110/112AA, D197A, was further reduced upon ectopic expression of HERV-K Gag versus MLV Gag (Fig. 6). However, the release efficiency of HIV-1 mutants VK181/182AA, WM184/185AA, LL189/190AA, K158A and P224A upon coexpression of HERV-K Gag was similar to that observed upon coexpression of MLV Gag (Fig. 6). These results suggest that intact HIV-1 CA-CTD or CA-CTD-mediated dimerization is prerequisite for inhibition by HERV-K Gag.

### HERV-K CA-MHR plays a key role in robust colocalization between HERV-K Gag and HIV-1 Gag at the plasma membrane

**Fig. 7:**
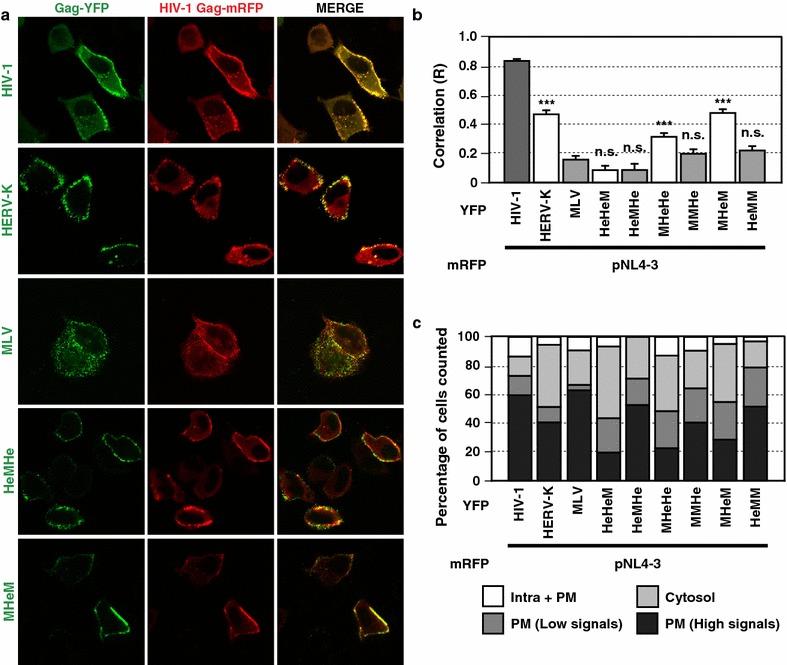
Most Gag chimeras containing HERV-K CA colocalize with HIV-1 Gag at the plasma membrane (PM). HeLa cells coexpressing YFP-tagged HERV-K Gag, MLV Gag or chimeric Gag (*green*) and mRFP-tagged HIV-1 Gag (*red*) proteins were examined using fluorescence microscopy at 16 h after cotransfection (**a**). Images acquired at the mid-section of the cells. **b** The R strength of correlation between fluorescence intensities of pairs of indicated Gag-fluorescent protein chimeras was calculated for cells coexpressing these Gag proteins. Data from 11 to 33 cells are shown as means ± standard error of the mean (SEM). *P* values were determined using a Student’s *t* test. *P < 0.01; ***P < 0.0001; *n.s.* not significant. **c** For the analysis of HIV-1 Gag localization patterns, we acquired images of 11–33 cells per condition at the middle and top focal plains. If Gag puncta distributes over the half of the circumference of a cell, the cell is classified as “PM (High signals)”. If Gag distributes less than the half of the cell circumference, the cell is classified as “PM (Low signals)”. If there is no Gag-puncta signal at the plasma membrane and in the cytosol in both top and middle focal plains of a cell, the cell is classified as “Cytosol”. If there is Gag puncta in the cytoplasm in the middle focal plain, it is classified as “Intra + PM”. Data from 11 to 33 cells are shown

**Fig. 8:**
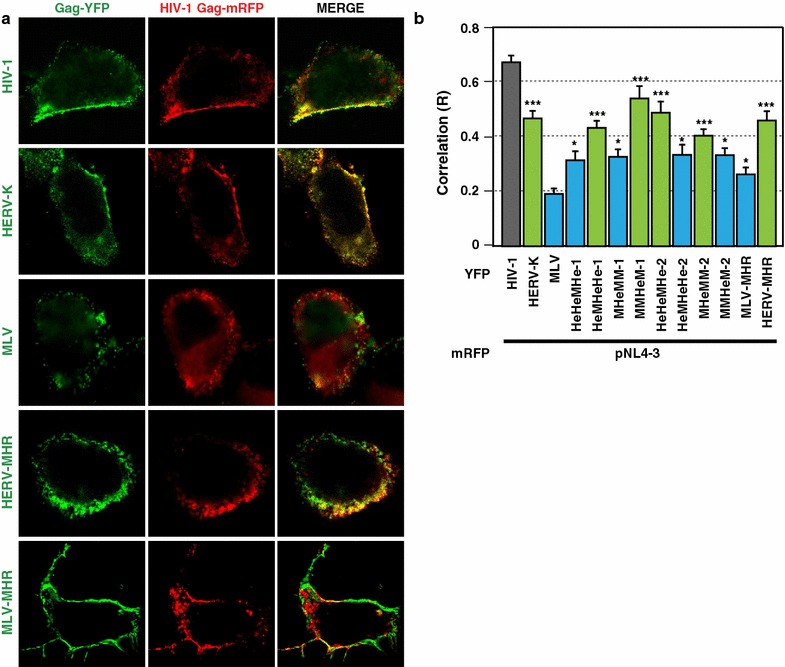
Chimeric Gag constructs containing a part of CA partially colocalize with HIV-1 Gag at the PM. HeLa cells coexpressing YFP-tagged HERV-K Gag, MLV Gag or chimeric Gag (*green*) and mRFP-tagged HIV-1 Gag (*red*) proteins were examined using fluorescence microscopy at 16 h after cotransfection (**a**). Images acquired at the mid-section of the cells. **b** The R strength of correlation between fluorescence intensities of pairs of indicated Gag-fluorescent protein chimeras was calculated for cells coexpressing these Gag proteins. *P* values were determined using a Student’s *t* test. *P < 0.01; ***P < 0.0001; *n.s.* not significant

To determine whether HERV-K Gag chimeras colocalize with HIV-1 Gag at the PM, HeLa cells were cotransfected with plasmids encoding YFP-tagged Gag chimera (or parental HERV-K or MLV Gag-YFP) and mRFP-tagged HIV-1 Gag. The localization of fluorescent-protein-tagged Gag proteins was determined using confocal microscopy 16 h post-transfection. As shown in previous studies, we observed punctate localization of HERV-K Gag-YFP at the PM and partial colocalization between HERV-K Gag-YFP and HIV-1 Gag-mRFP (Figs. 7a, 8a). In contrast, MLV Gag-YFP did not colocalize with HIV-1 Gag-mRFP (Figs. 7a, 8a), as observed in previous reports [18, 55]. Therefore, we predicted that YFP-tagged constructs of Gag chimeras that efficiently inhibit HIV-1 release (e.g., MHeM) would colocalize with HIV-1 Gag-mRFP more extensively than those that do not (e.g., HeMHe). As expected, MHeHe and MHeM Gag-YFP colocalized with HIV-1 Gag-mRFP, whereas HeMHe Gag-YFP did not colocalize with HIV-1 Gag-mRFP (Fig. 7a, b and Additional file 2: Figure S2A). However, HeHeM Gag-YFP did not colocalize with HIV-1 Gag-mRFP despite its strong inhibitory effect on HIV-1 release (Fig. 3). It appears that HeHeM Gag-YFP coexpression efficiently increases HIV-1 Gag localization in the cytosol and decreases its PM localization (Fig. 7c). Therefore, HIV-1 Gag that formed multimers with HeHeM Gag might detach from the PM and accumulated in the cytosol. While membrane binding of HERV-K Gag is necessary for inhibition of HIV-1 release [18], at this point we do not rule out the possibility that HeHeM Gag-YFP may be able to suppress HIV-1 assembly without or prior to colocalization with HIV-1 Gag at the PM. To further determine the important region in the HERV-K CA domain for colocalization with HIV-1 Gag, HeLa cells were cotransfected with YFP-tagged CA chimeras and HIV-1 Gag-mRFP (Fig. 8a, b and Additional file 2: Figure S2B). Unexpectedly, all of these Gag-YFP chimeras colocalized with HIV-1 Gag-mRFP more efficiently than MLV Gag-YFP (Fig. 8a, b and Additional file 2: Figure S2B). Among them, HeHeMHe-1 and MHeMM-1, both of which encode CA-NTD of HERV-K Gag, as well as HeMHeHe-2 and MMHeM-2, showed only intermediate colocalization with HIV-1 Gag. On the other hand, HeMHeHe-1, MMHeM-1, HeHeMHe-2 and MHeMM-2, all of which encode HERV-K CA-MHR, colocalize as efficiently with HIV-1 Gag-mRFP as HERV-K Gag regardless of the origin of CA-NTD. Furthermore, substitution of CA-MHR alone allowed efficient colocalization of MLV Gag with HIV-1 Gag (Fig. 8a, b). These results suggest that HERV-K Gag CA-MHR promotes colocalization between HIV-1 Gag and HERV-K Gag; however, both HERV-K CA-NTD and the region downstream of MHR are capable of mediating partial colocalization with HIV-1 Gag. Notably, colocalization efficiency does not fully correlate with the efficiency of HIV-1 release inhibition. For chimeric Gag proteins encoding HERV-K CA-NTD, only the partial colocalization appears to be enough for the reduction of HIV-1 release.

### HERV-K CA-NTD and MHR are not required for reduction of HIV-1 infectivity

**Fig. 9:**
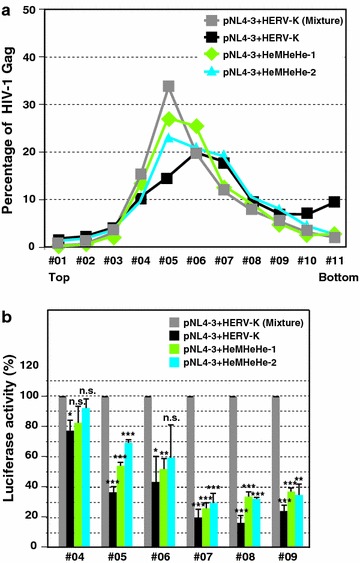
HERV-K CA-NTD and MHR are not required for reduction of HIV-1 infectivity. HeLa cells were cotransfected with pNL4-3 and indicated plasmids. **a** Virus in the supernatant from cotransfected HeLa cells were collected and fractionated in rate-zonal gradient analysis. The amount of HIV-1 Gag were measured by ELISA. **b** Virus stocks were prepared from each fraction after rate-zonal gradient analysis. The viruses were purified and normalized by p24 ELISA. TZM-bl cells, which harbor an HIV-1 LTR-driven luciferase-reporter gene, were infected with the purified viruses. At 2 days post-infection, luciferase activities were measured by luminometor. Data from three independent experiments are shown as means ± standard deviations. *P* values were determined using a Student’s *t* test. *P < 0.01; **P < 0.001; ***P < 0.0001; *n.s.* not significant

To test whether the same region of HERV-K Gag is responsible for inhibition of HIV-1 release and virion infectivity, we compared HeMHeHe-1 and -2 with WT HERV-K Gag for the effects on HIV-1 particle properties. Upon coexpression of the chimeras, the peak of HIV-1 Gag p24 in the rate zonal gradient analysis shifted toward bottom compared to when HIV-1 is singly expressed although the shift was smaller than that observed in the presence of WT HERV-K Gag (Fig. 9a). These results suggest that HERV-K CA-NTD is not absolutely required for changing the property of HIV-1 particles.

To examine the infectivity of HIV-1 particles with the larger size, we purified the fractionated viruses from sucrose gradients. The purified virus in each fraction was normalized by amounts of p24 antigens. TZM-bl cells, which encodes Tat-driven luciferase gene, were infected with these normalized viruses. The infectivity of HIV-1 in the fraction #04 was not severely reduced by HERV-K Gag (Fig. 9b). However, the infectivity of HIV-1 in the fractions #07-09, which are likely to be enriched in coassembled particles, was drastically reduced (Fig. 9b). Chimeric HERV-K Gags encoding MLV Gag CA-NTD reduced the infectivity of HIV-1 similarly. These results suggest that HERV-K CA-NTD is not necessary for inhibition of HIV-1 infectivity (Fig. 9b). In addition, the presence of HERV-K CA MHR, which is important for extensive colocalization with HIV-1 Gag (Fig. 8), did not exacerbate the infectivity defect of HIV-1 virions, suggesting that HERV-K CA MHR and the high-level colocalization dependent on this region are dispensable for inhibition of HIV-1 infectivity.

## Discussion

In this study, we found that HERV-K Gag CA-NTD is important for efficient inhibition of HIV-1 release, whereas CA-MHR is required for higher colocalization between HIV-1 Gag and HERV-K Gag, and yet both CA-NTD and CA-MHR are not essential for inhibition of infectivity. These findings suggest that HERV-K Gag inhibits HIV-1 release and impairs infectivity of released progeny virions in genetically separable mechanisms.

HERV-K Gag consists of 4 major domains, MA, p15, CA and NC domains. Previously, we suggested that HERV-K Gag coassembles with HIV-1 Gag at the PM in a manner dependent on MA-mediated membrane binding and NC-mediated RNA binding [18]. However, MLV Gag, which binds to the PM and RNA just like HERV-K Gag, did not colocalize or coassemble with HIV-1 Gag and failed to inhibit HIV-1 release. It was unclear how HERV-K Gag, but not MLV Gag, targets to the assembly sites of HIV-1 Gag at the PM. In this study, we found that HERV-K CA, when replaced with MLV CA, can promote colocalization of MLV Gag with HIV-1 Gag at the PM in most cases (Fig. 7b). Among the tested HERV-K CA regions, MHR promotes the colocalization most efficiently (Fig. 8b). Nonetheless, it appears that any of the tested HERV-K CA regions is capable of promoting colocalization with HIV-1 Gag.

We observed that a single chimera, HeHeM, showed minimal correlation of distribution with HIV-1 Gag despite containing the entire HERV-K CA. It is possible that this chimera suppresses the HIV-1 assembly process at the step of membrane binding of Gag multimer. We speculate that heteromultimerization between HIV-1 Gag and HeHeM Gag might destabilize PM binding of the Gag multimer at an early step of HIV-1 assembly (see below), prevent formation of prominent coassemblies at the PM, and thereby reduce colocalization between HIV-1 Gag and the chimera. In this regard, it is of note that the colocalization efficiency represented by the R value may underestimate the ability of Gag constructs to heteromultimerize with HIV-1 Gag if a large fraction of cells display only cytosolic HIV-1 Gag as observed in cultures coexpressing WT HERV-K Gag or chimeras containing the entire HERV-K CA.

Based on TSG101 experiments, coassembly of HERV-K Gag is likely to take place at the early stage of HIV-1 Gag assembly (Fig. 5). The Lingappa group reported that three classes of HIV-1 CA residues are involved in distinct steps of virus assembly [52, 56]. Six residues in CA-CTD, VK181/182, WM184/185 and LL189/190, are involved in the dimerization of CA-CTD. Three residues in CA-CTD, K158, D197 and P224, are involved in the low order multimerization. Finally, eight residues, EE75/76, RS100/102, TT107/108 and TQ110/112 in CA-NTD are important for the completion of particle formation. In this study, we found that release efficiency of 5 HIV-1 mutants, VK181/182AA, WM184/185AA, LL189/190AA, K158A and P224A is not reduced by HERV-K Gag (Fig. 6). Therefore, the early assembly steps, which require Gag dimerization or low-order multimerization, are likely to be the target of HERV-K Gag or prerequisite for the process inhibited by HERV-K Gag. It is unclear why the virus release efficiency of D197A mutant was reduced by HERV-K Gag unlike that of K158A or P224A mutants. However, we observed that the D197A mutant releases 10-fold more p24 than K158A and P224A mutants in our experiments (Fig. 6). Therefore, it is possible that D197A might impose less severe suppression than K158A or P224A on the stage susceptible to the inhibition by HERV-K. We also do not rule out an alternative possibility that VK181/182AA, WM184/185AA, LL189/190AA, K158A and P224A may directly or indirectly disrupt the interface for HERV-K Gag.

We observed that coexpression of HERV-K Gag reduces the number of HIV-1 particles with mature core formation (Fig. 1c, e) although it did not cause accumulation of HIV-1 Pr55 Gag in virions (Fig. 3b). In this regard, the effect of HERV-K Gag is reminiscent of the effect of bevirimat, a maturation inhibitor, except that the CA-SP1 fragment was not detected in either cells or viruses unlike with the case with bevirimat treatment. Indeed, the morphology of HIV-1 virions produced from cells coexpressing HERV-K Gag (frame 2 of Fig. 1c) appears similar to that of HIV-1 particles produced by cells treated with bevirimat [57–59]. Notably, within HERV-K CA regions, both CA-NTD and MHR are not required for impairing the infectivity of HIV-1 (Fig. 9b). Therefore, it is possible that HERV-K Gag impairs HIV-1 core formation or stability after the processing of HIV-1 Gag and does so by interactions with HIV CA via its CA C-terminal region outside of MHR. Further studies focused on the morphology and biochemical properties of cores of co-assembled particles containing HERV-K CA chimeras should help establish the relationship between HERV-K Gag-induced changes in HIV-1 maturation and impairment of its infectivity.

Previous studies observed that HERV-K expression is increased upon HIV-1 infection in T cells [33, 39]. Consistent with this, we observed HERV-K induction by HIV-1 infection in an in vitro experiment (data not shown). It is tempting to suggest that HERV-K sequences have existed in the human genome under several selection pressures and might have protected the host cells from the threat of exogenous viruses as are the case with other endogenized viruses. Fv1, which is a remnant of mouse endogenous retrovirus Gag, interferes with the post-entry process of MLV infection [60–63]. Endogenous retroelements Fv4, enJSRV Env and Refrex-1 Env prevent the entry of exogenous retroviruses MLV [64], JSRV [65] and FeLV-2 [66, 67], respectively, through receptor masking. Similar to HERV-K [18], enJSRV also blocks the JSRV particle formation via its Gag [65]. Recently, Wysocka group reported that HERV-K element (Rec), which is expressed in early embryogenesis, appears to induce an innate immune response (IFITM1) and protect the host cells from exogenous viral infection [23]. In the current study, we showed that HERV-K Gag can suppress the HIV-1 assembly at an early step and alter the properties of HIV-1 particles, via distinct molecular mechanisms. Altogether, endogenous retroviral elements are likely to have been contributing survival of the hosts in the evolutionary time scale via a wide variety of mechanisms.

## Conclusions

HERV-K is principally expressed in germ cells, but eventually silenced through the development process. Interestingly, however, accumulating evidence suggests that HERV-K reappears in HIV-1-infected patients. We previously reported that HERV-K Gag coassembles with HIV-1 Gag and interferes with the HIV-1 release. Moreover, HERV-K Gag reduces the infectivity of HIV-1. However, the molecular mechanisms by which HERV-K Gag interferes with HIV-1 replication remain poorly understood. In this study, we found that HERV-K CA domain is important for specific incorporation into HIV-1 virions and reduction of HIV-1 release. HERV-K Gag interfered with HIV-1 Gag assembly at an early step(s) and changed HIV-1 particle properties including core formation and infectivity. The effects on HIV-1 release and infectivity were genetically separable.

## Additional files

**Additional file 1: Fig. S1.** Coexpression of HERV-K GagPro reduces HIV-1 release efficiency and infectivity of released particles. HeLa cells were cotransfected with HIV-1 pNL4-3 and indicated plasmids encoding non-Flag-tagged Gag at different ratios (A) and at 10:1 ratio (C). The amount of p24 was measured as described in Fig. 3c. *P* values, compared with HERV-K Gag, were determined using a Student’s *t* test. *, *P* < 0.01; **, *P* < 0.001; ***, *P* < 0.0001; n.s., not significant. (B) Cell and viral lysates from cotransfected cells were subjected to SDS-PAGE and analyzed by immunoblotting with HIV-Ig or anti-HERV-K Gag antibody. (D) The viruses were recovered from supernatants by ultracentrifugation and normalized by p24 ELISA. TZM-bl cells were infected with the recovered viruses. At 2 days post-infection, luciferase activities were measured by luminometor. Data from three independent experiments are shown as means ± standard deviations. *P* values were determined using a Student’s *t* test. *, *P* < 0.01; **, *P* < 0.001; ***, *P* < 0.0001; n.s., not significant.

**Additional file 2: Fig. S2.** Chimeric Gag constructs containing a part of HERV-K CA colocalize at least partially with HIV-1 Gag at the PM. HeLa cells coexpressing YFP-tagged chimeric Gag (green) and mRFP-tagged HIV-1 Gag (red) proteins were examined using fluorescence microscopy at 16 h after cotransfection (A and B). Images were acquired at the mid-section of the cells.

## References

[1] Bannert N, Kurth R (2004). Retroelements and the human genome: new perspectives on an old relation. Proc Natl Acad Sci USA.

[2] Lander ES, Linton LM, Birren B, Nusbaum C, Zody MC, Baldwin J (2001). Initial sequencing and analysis of the human genome. Nature.

[3] Venter JC, Adams MD, Myers EW, Li PW, Mural RJ, Sutton GG (2001). The sequence of the human genome. Science.

[4] Boeke JD, Stoye JP. Retrotransposons, endogenous retroviruses, and the evolution of retroelements. In: Coffin JM, Hughes SH, Varmus HE, editors. Retroviruses; 1997. pp. 343–436. https://books.google.co.jp/books?id=x6XuCAAAQBAJ&pg=PA92&lpg=PA92&dq=Retrotransposons,+Endogenous+Retroviruses,+and+the+Evolution+of+Retroelements.&source=bl&ots=xHgMSbMe9v&sig=nKT0ev4jkjvEIRiCM0OJv5OKeX8&ZvhbLTAhWCvrwKHQZvCX4Q6AEIQDAD#v=onepage&q=Retrotransposons%2C%20Endogenous%20Retroviruses%2C%20and%20the%20Evolution%20of%20Retroelements.&f=false. 21433351

[5] Stoye JP (2012). Studies of endogenous retroviruses reveal a continuing evolutionary saga. Nat Rev Microbiol.

[6] Turner G, Barbulescu M, Su M, Jensen-Seaman MI, Kidd KK, Lenz J (2001). Insertional polymorphisms of full-length endogenous retroviruses in humans. Curr Biol.

[7] Beimforde N, Hanke K, Ammar I, Kurth R, Bannert N (2008). Molecular cloning and functional characterization of the human endogenous retrovirus K113. Virology.

[8] Boller K, Schonfeld K, Lischer S, Fischer N, Hoffmann A, Kurth R (2008). Human endogenous retrovirus HERV-K113 is capable of producing intact viral particles. J Gen Virol.

[9] Lee YN, Bieniasz PD (2007). Reconstitution of an infectious human endogenous retrovirus. PLoS Pathog.

[10] Dewannieux M, Harper F, Richaud A, Letzelter C, Ribet D, Pierron G (2006). Identification of an infectious progenitor for the multiple-copy HERV-K human endogenous retroelements. Genome Res.

[11] Ono A (2010). HIV-1 assembly at the plasma membrane. Vaccine.

[12] Adamson CS, Freed EO (2007). Human immunodeficiency virus type 1 assembly, release, and maturation. Adv Pharmacol.

[13] Sundquist WI, Krausslich HG (2012). HIV-1 assembly, budding, and maturation. Cold Spring Harb Perspect Med.

[14] Bieniasz PD (2009). The cell biology of HIV-1 virion genesis. Cell Host Microbe.

[15] Balasubramaniam M, Freed EO (2011). New insights into HIV assembly and trafficking. Physiology (Bethesda).

[16] George M, Schwecke T, Beimforde N, Hohn O, Chudak C, Zimmermann A (2011). Identification of the protease cleavage sites in a reconstituted Gag polyprotein of an HERV-K(HML-2) element. Retrovirology.

[17] Kraus B, Boller K, Reuter A, Schnierle BS (2011). Characterization of the human endogenous retrovirus K Gag protein: identification of protease cleavage sites. Retrovirology.

[18] Monde K, Contreras-Galindo R, Kaplan MH, Markovitz DM, Ono A (2012). Human endogenous retrovirus K Gag coassembles with HIV-1 Gag and reduces the release efficiency and infectivity of HIV-1. J Virol.

[19] Chudak C, Beimforde N, George M, Zimmermann A, Lausch V, Hanke K (2013). Identification of late assembly domains of the human endogenous retrovirus-K(HML-2). Retrovirology.

[20] Heslin DJ, Murcia P, Arnaud F, Van Doorslaer K, Palmarini M, Lenz J (2009). A single amino acid substitution in a segment of the CA protein within Gag that has similarity to human immunodeficiency virus type 1 blocks infectivity of a human endogenous retrovirus K provirus in the human genome. J Virol.

[21] Bieda K, Hoffmann A, Boller K (2001). Phenotypic heterogeneity of human endogenous retrovirus particles produced by teratocarcinoma cell lines. J Gen Virol.

[22] Herbst H, Sauter M, Mueller-Lantzsch N (1996). Expression of human endogenous retrovirus K elements in germ cell and trophoblastic tumors. Am J Pathol.

[23] Grow EJ, Flynn RA, Chavez SL, Bayless NL, Wossidlo M, Wesche DJ (2015). Intrinsic retroviral reactivation in human preimplantation embryos and pluripotent cells. Nature.

[24] Muster T, Waltenberger A, Grassauer A, Hirschl S, Caucig P, Romirer I (2003). An endogenous retrovirus derived from human melanoma cells. Cancer Res.

[25] Contreras-Galindo R, Kaplan MH, Leissner P, Verjat T, Ferlenghi I, Bagnoli F (2008). Human endogenous retrovirus K (HML-2) elements in the plasma of people with lymphoma and breast cancer. J Virol.

[26] Lower R, Lower J, Kurth R (1996). The viruses in all of us: characteristics and biological significance of human endogenous retrovirus sequences. Proc Natl Acad Sci USA.

[27] Vogetseder W, Dumfahrt A, Mayersbach P, Schonitzer D, Dierich MP (1993). Antibodies in human sera recognizing a recombinant outer membrane protein encoded by the envelope gene of the human endogenous retrovirus K. AIDS Res Hum Retrovir.

[28] Garrison KE, Jones RB, Meiklejohn DA, Anwar N, Ndhlovu LC, Chapman JM (2007). T cell responses to human endogenous retroviruses in HIV-1 infection. PLoS Pathog.

[29] SenGupta D, Tandon R, Vieira RG, Ndhlovu LC, Lown-Hecht R, Ormsby CE (2011). Strong human endogenous retrovirus-specific T cell responses are associated with control of HIV-1 in chronic infection. J Virol.

[30] Tandon R, SenGupta D, Ndhlovu LC, Vieira RG, Jones RB, York VA (2011). Identification of human endogenous retrovirus-specific T cell responses in vertically HIV-1-infected subjects. J Virol.

[31] Contreras-Galindo R, Gonzalez M, Almodovar-Camacho S, Gonzalez-Ramirez S, Lorenzo E, Yamamura Y (2006). A new Real-Time-RT-PCR for quantitation of human endogenous retroviruses type K (HERV-K) RNA load in plasma samples: increased HERV-K RNA titers in HIV-1 patients with HAART non-suppressive regimens. J Virol Methods.

[32] Contreras-Galindo R, Kaplan MH, Markovitz DM, Lorenzo E, Yamamura Y (2006). Detection of HERV-K(HML-2) viral RNA in plasma of HIV type 1-infected individuals. AIDS Res Hum Retrovir.

[33] Contreras-Galindo R, Lopez P, Velez R, Yamamura Y (2007). HIV-1 infection increases the expression of human endogenous retroviruses type K (HERV-K) in vitro. AIDS Res Hum Retrovir.

[34] Contreras-Galindo R, Kaplan MH, Contreras-Galindo AC, Gonzalez-Hernandez MJ, Ferlenghi I, Giusti F (2012). Characterization of human endogenous retroviral elements in the blood of HIV-1-infected individuals. J Virol.

[35] Li SK, Leung RK, Guo HX, Wei JF, Wang JH, Kwong KT (2012). Detection and identification of plasma bacterial and viral elements in HIV/AIDS patients in comparison to healthy adults. Clin Microbiol Infect.

[36] Lefebvre G, Desfarges S, Uyttebroeck F, Munoz M, Beerenwinkel N, Rougemont J (2011). Analysis of HIV-1 expression level and sense of transcription by high-throughput sequencing of the infected cell. J Virol.

[37] Contreras-Galindo R, Kaplan MH, He S, Contreras-Galindo AC, Gonzalez-Hernandez MJ, Kappes F (2013). HIV infection reveals widespread expansion of novel centromeric human endogenous retroviruses. Genome Res.

[38] Gonzalez-Hernandez MJ, Cavalcoli JD, Sartor MA, Contreras-Galindo R, Meng F, Dai M (2014). Regulation of the human endogenous retrovirus K (HML-2) transcriptome by the HIV-1 Tat protein. J Virol.

[39] Gonzalez-Hernandez MJ, Swanson MD, Contreras-Galindo R, Cookinham S, King SR, Noel RJ (2012). Expression of human endogenous retrovirus type K (HML-2) is activated by the Tat protein of HIV-1. J Virol.

[40] Sun Z, Pan J, Hope WX, Cohen SN, Balk SP (1999). Tumor susceptibility gene 101 protein represses androgen receptor transactivation and interacts with p300. Cancer.

[41] Platt EJ, Wehrly K, Kuhmann SE, Chesebro B, Kabat D (1998). Effects of CCR5 and CD4 cell surface concentrations on infections by macrophagetropic isolates of human immunodeficiency virus type 1. J Virol.

[42] Wei X, Decker JM, Liu H, Zhang Z, Arani RB, Kilby JM (2002). Emergence of resistant human immunodeficiency virus type 1 in patients receiving fusion inhibitor (T-20) monotherapy. Antimicrob Agents Chemother.

[43] Garnier L, Parent LJ, Rovinski B, Cao SX, Wills JW (1999). Identification of retroviral late domains as determinants of particle size. J Virol.

[44] Gonda MA, Aaronson SA, Ellmore N, Zeve VH, Nagashima K (1976). Ultrastructural studies of surface features of human normal and tumor cells in tissue culture by scanning and transmission electron microscopy. J Natl Cancer Inst.

[45] Demirov DG, Ono A, Orenstein JM, Freed EO (2002). Overexpression of the N-terminal domain of TSG101 inhibits HIV-1 budding by blocking late domain function. Proc Natl Acad Sci USA.

[46] Chu HH, Chang YF, Wang CT (2006). Mutations in the alpha-helix directly C-terminal to the major homology region of human immunodeficiency virus type 1 capsid protein disrupt Gag multimerization and markedly impair virus particle production. J Biomed Sci.

[47] Gamble TR, Yoo S, Vajdos FF, von Schwedler UK, Worthylake DK, Wang H (1997). Structure of the carboxyl-terminal dimerization domain of the HIV-1 capsid protein. Science.

[48] Joshi A, Nagashima K, Freed EO (2006). Mutation of dileucine-like motifs in the human immunodeficiency virus type 1 capsid disrupts virus assembly, gag-gag interactions, gag-membrane binding, and virion maturation. J Virol.

[49] Klein KC, Reed JC, Tanaka M, Nguyen VT, Giri S, Lingappa JR (2011). HIV Gag-leucine zipper chimeras form ABCE1-containing intermediates and RNase-resistant immature capsids similar to those formed by wild-type HIV-1 Gag. J Virol.

[50] Ono A, Waheed AA, Joshi A, Freed EO (2005). Association of human immunodeficiency virus type 1 gag with membrane does not require highly basic sequences in the nucleocapsid: use of a novel Gag multimerization assay. J Virol.

[51] von Schwedler UK, Stray KM, Garrus JE, Sundquist WI (2003). Functional surfaces of the human immunodeficiency virus type 1 capsid protein. J Virol.

[52] Robinson BA, Reed JC, Geary CD, Swain JV, Lingappa JR (2014). A temporospatial map that defines specific steps at which critical surfaces in the Gag MA and CA domains act during immature HIV-1 capsid assembly in cells. J Virol.

[53] Hogue IB, Grover JR, Soheilian F, Nagashima K, Ono A (2011). Gag induces the coalescence of clustered lipid rafts and tetraspanin-enriched microdomains at HIV-1 assembly sites on the plasma membrane. J Virol.

[54] Grover JR, Llewellyn GN, Soheilian F, Nagashima K, Veatch SL, Ono A (2013). Roles played by capsid-dependent induction of membrane curvature and Gag-ESCRT interactions in tetherin recruitment to HIV-1 assembly sites. J Virol.

[55] Ako-Adjei D, Johnson MC, Vogt VM (2005). The retroviral capsid domain dictates virion size, morphology, and coassembly of gag into virus-like particles. J Virol.

[56] Tanaka M, Robinson BA, Chutiraka K, Geary CD, Reed JC, Lingappa JR (2015). Mutations of conserved residues in the major homology region arrest assembling HIV-1 Gag as a membrane-targeted intermediate containing genomic RNA and cellular proteins. J Virol.

[57] Keller PW, Adamson CS, Heymann JB, Freed EO, Steven AC (2011). HIV-1 maturation inhibitor bevirimat stabilizes the immature Gag lattice. J Virol.

[58] Li F, Goila-Gaur R, Salzwedel K, Kilgore NR, Reddick M, Matallana C (2003). PA-457: a potent HIV inhibitor that disrupts core condensation by targeting a late step in Gag processing. Proc Natl Acad Sci USA.

[59] Wiegers K, Rutter G, Kottler H, Tessmer U, Hohenberg H, Krausslich HG (1998). Sequential steps in human immunodeficiency virus particle maturation revealed by alterations of individual Gag polyprotein cleavage sites. J Virol.

[60] Jolicoeur P, Baltimore D (1976). Effect of Fv-1 gene product on proviral DNA formation and integration in cells infected with murine leukemia viruses. Proc Natl Acad Sci USA.

[61] Sveda MM, Soeiro R (1976). Host restriction of Friend leukemia virus: synthesis and integration of the provirus. Proc Natl Acad Sci USA.

[62] Jern P, Coffin JM (2008). Effects of retroviruses on host genome function. Annu Rev Genet.

[63] Best S, Le Tissier P, Towers G, Stoye JP (1996). Positional cloning of the mouse retrovirus restriction gene Fv1. Nature.

[64] Kozak CA, Gromet NJ, Ikeda H, Buckler CE (1984). A unique sequence related to the ecotropic murine leukemia virus is associated with the Fv-4 resistance gene. Proc Natl Acad Sci USA.

[65] Palmarini M, Mura M, Spencer TE (2004). Endogenous betaretroviruses of sheep: teaching new lessons in retroviral interference and adaptation. J Gen Virol.

[66] Ito J, Watanabe S, Hiratsuka T, Kuse K, Odahara Y, Ochi H (2013). Refrex-1, a soluble restriction factor against feline endogenous and exogenous retroviruses. J Virol.

[67] Ito J, Baba T, Kawasaki J, Nishigaki K (2016). Ancestral mutations acquired in Refrex-1, a restriction factor against feline retroviruses, during its cooption and domestication. J Virol.

