# Supplementary figures and images for: Molecular mechanisms by which HERV-K Gag interferes with HIV-1 Gag assembly and particle infectivity
Source: Retrovirology. 2017 Apr 26;14:27. doi: 10.1186/s12977-017-0351-8 (PMC5406883; doi:10.1186/s12977-017-0351-8)

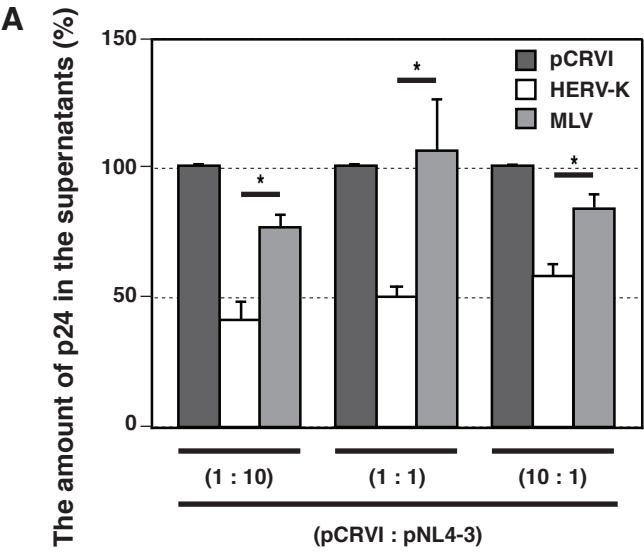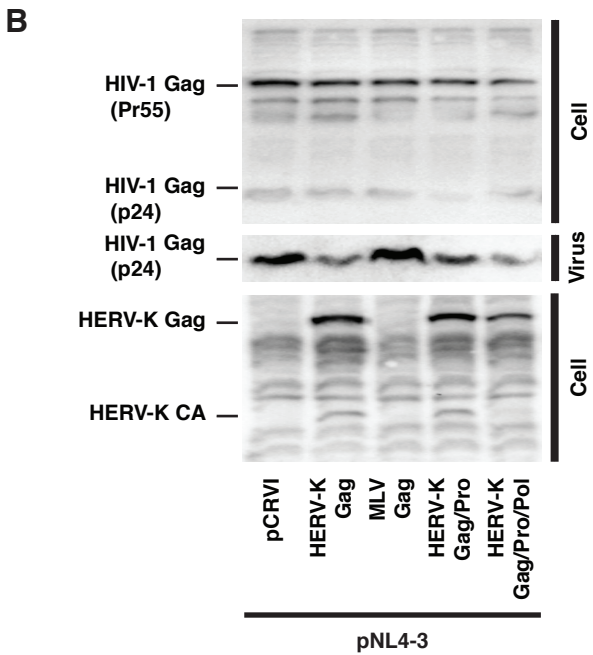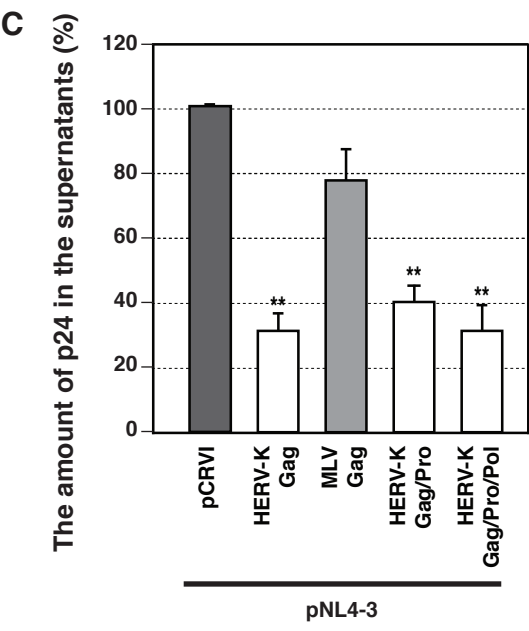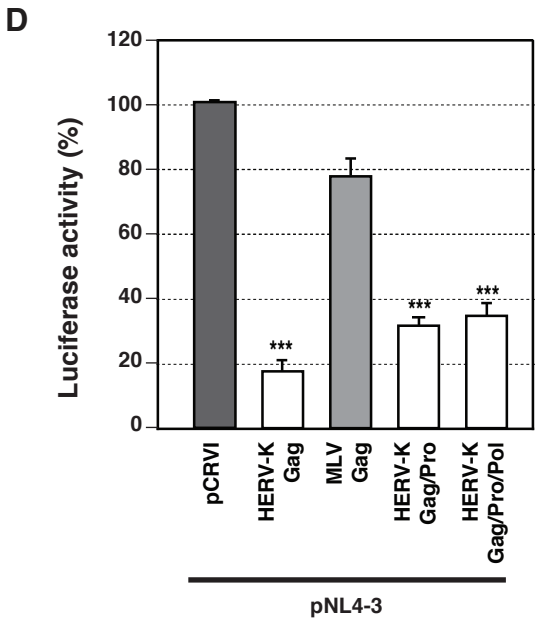

Supplement: Supplementary file 1 — Additional file 1: Fig. S1. Coexpression of HERV-K GagPro reduces HIV-1 release efficiency and infectivity of released particles. HeLa cells were cotransfected with HIV-1 pNL4-3 and indicated plasmids encoding non-Flag-tagged Gag at different ratios (A) and at 10:1 ratio (C). The amount of p24 was measured as described in Fig. 3c. P values, compared with HERV-K Gag, were determined using a Student’s t test. *, P < 0.01; **, P < 0.001; ***, P < 0.0001; n.s., not significant. (B) Cell and viral lysates from cotransfected cells were subjected to SDS-PAGE and analyzed by immunoblotting with HIV-Ig or anti-HERV-K Gag antibody. (D) The viruses were recovered from supernatants by ultracentrifugation and normalized by p24 ELISA. TZM-bl cells were infected with the recovered viruses. At 2 days post-infection, luciferase activities were measured by luminometor. Data from three independent experiments are shown as means ± standard deviations. P values were determined using a Student’s t test. *, P < 0.01; **, P < 0.001; ***, P < 0.0001; n.s., not significant. [file 12977_2017_351_MOESM1_ESM.pdf]

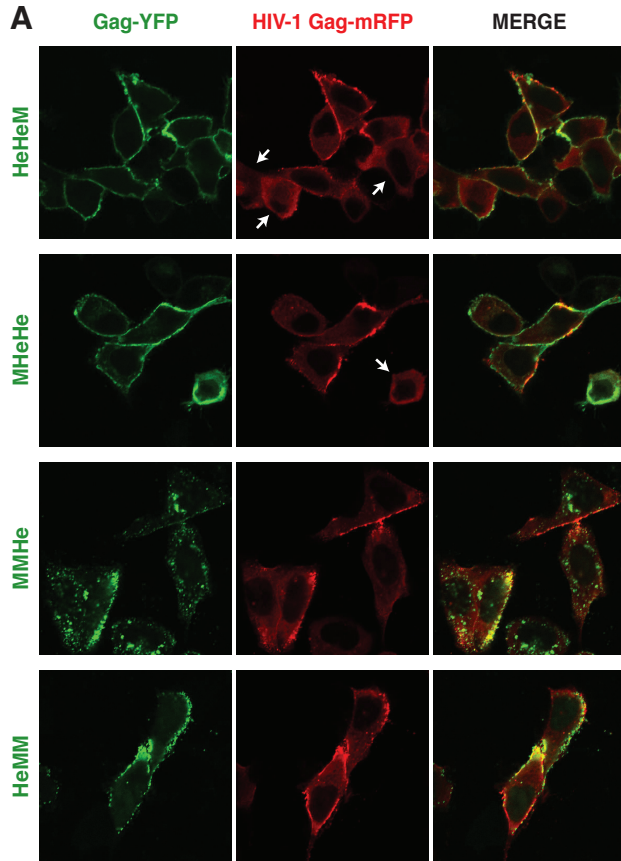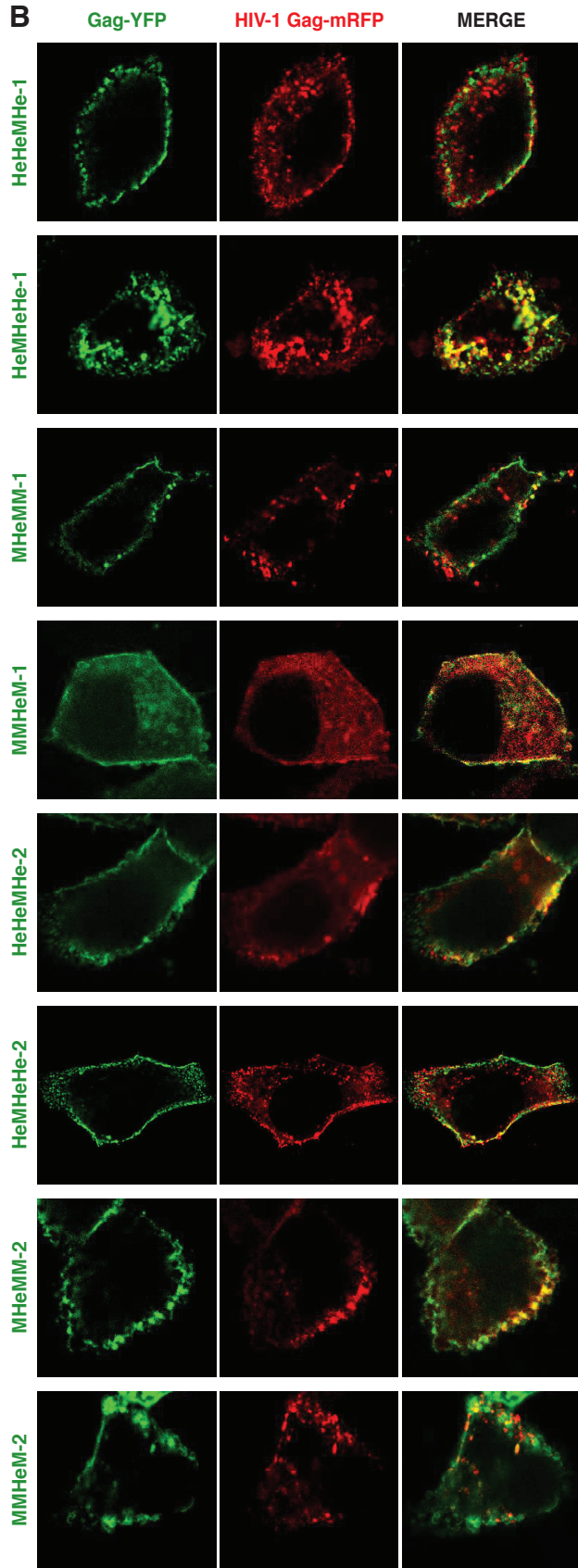

Supplement: Supplementary file 2 — Additional file 2: Fig. S2. Chimeric Gag constructs containing a part of HERV-K CA colocalize at least partially with HIV-1 Gag at the PM. HeLa cells coexpressing YFP-tagged chimeric Gag (green) and mRFP-tagged HIV-1 Gag (red) proteins were examined using fluorescence microscopy at 16 h after cotransfection (A and B). Images were acquired at the mid-section of the cells. [file 12977_2017_351_MOESM2_ESM.pdf]
